# Nedd4l downregulation of NRG1 in the mPFC induces depression-like behaviour in CSDS mice

**DOI:** 10.1038/s41398-020-00935-x

**Published:** 2020-07-23

**Authors:** Jia Xu, Cuiping Guo, Yi Liu, Gang Wu, Dan Ke, Qun Wang, Jing Mao, Jian-Zhi Wang, Rong Liu, Xiaochuan Wang

**Affiliations:** 1 Department of Pathophysiology, School of Basic Medicine, Key Laboratory of Education Ministry of China for Neurological Disorders, Tongji Medical College, Huazhong University of Science and Technology, Wuhan, 430030 China; 2 Department of Pathophysiology, Weifang Medical University, Weifang, 261053 China; 3 School of Nursing, Tongji Medical College, Huazhong University of Science and Technology, Wuhan, 430030 China; 4 Co-innovation Center of Neuroregeneration, Nantong University, Nantong, Jiangsu 226001 China

**Keywords:** Psychology, Depression

## Abstract

The occurrence of major depressive disorders has been closely related to the vulnerability of stress. The medial prefrontal cortex (mPFC) is involved in regulating pathological reactivity to stress, changes in affective behaviour and cognitive functions by distress. Increasing evidence indicates that neuregulin 1 (NRG1) plays an important role in psychiatric illnesses, including depression, schizophrenia and bipolar disorder. However, whether NRG1 in the mPFC is related to stress vulnerability remains unclear. We here assessed the regulation of NRG1 by the E3 ubiquitin ligase Nedd4l (neural precursor cell expressed developmentally downregulated 4-like) and investigated whether NRG1 changes in the mPFC might lead to vulnerability to depression-like behaviours. We’ve identified a deficiency of NRG1 in the mPFC as a key factor that contributes to the regulation of stress susceptibility in mice, as further suggested by the finding that overexpression of NRG1 attenuated depression-like behaviours in the animal model of chronic social defeat stress (CSDS). Interestingly, RNA sequencing in the mPFC brain region showed no differences in NRG1 mRNA levels between control animals and stress-susceptible (SS) or resilient mice (RES) following CSDS. However, mRNA and protein levels of Nedd4l were markedly increased in SS mice, but not in RES mice compared to controls. Furthermore, ubiquitination of NRG1 was increased in SS mice. Remarkably, overexpression of Nedd4l in mouse mPFC induced a decrease in NRG1 level and caused vulnerability to stress by subthreshold social defeat stress (SSDS), while downregulation of Nedd4l expression in the mPFC rescued the vulnerability to stress-induced social avoidance and anhedonia. Our data strongly indicate that the Nedd4l-mediated downregulation of NRG1 acts as a critical role in depression-like phenotypes of mice in CSDS.

## Introduction

Major depressive disorder (MDD) is a common mental illness. Nowadays, interpersonal stress and chronic social rejection have been regarded as the strongest risk factors for depression^1^. The most common time of onset is in a person’s 20s and 30s. Therefore, MDD is becoming a significant problem affecting young people’s health^2^. Basic and clinical studies have demonstrated that depression is related to a reduction in the size and neuronal synapse density of the prefrontal cortex and the hippocampus^3^. Social defeat is one source of chronic mental illness^4^, and causes major changes in brain functioning, neurotransmitters, hormone levels behaviour and health^5^. At the present, the chronic social defeat stress (CSDS) is a common paradigm to investigate the effect of social stress on depression-like behaviour in mice^6,7^.

Neuregulin 1 (NRG1) is a kind of epidermal growth factor-like proteins^8^ that are largely distributed in the frontal cortex, cerebellum and midbrain, and interact with the ErbB family to be involved in neurodevelopment and synaptic plasticity^9^. NRG1 deficiency within the cortical projection neurons resulted in increased inhibitory connections and reduced synaptic plasticity^10,11^. Also, chronic stress leads to a decrease in spine density in the cortex^12^. Especially, the medial prefrontal cortex (mPFC) is one among the critical brain regions involved within regulating the pathological reactivity to stress^13^, changes in affective behaviour, neuroendocrine response and cognition that are negatively affected by chronic stress^14^. However, the molecular mechanism by which chronic stress affects the neural representation of behaviour in the mPFC remains elusive.

E3 ligases have been reported to regulate the levels of EGFR family receptors^15,16^. As a highly conserved E3 ligase, Nedd4l (neural precursor cell expressed developmentally downregulated 4-like) is referred to as Nedd4-2, which is largely expressed in the mouse brain^17,18^.

Using RNA sequencing (RNA-seq) and examining brain tissue samples, we observed that the mRNA and protein levels of Nedd4l were obviously increased in the mPFC brain region of stress-susceptible (SS) mice. At the same time, the protein level of NRG1 was reduced in SS mice, but the mRNA level of NRG1 remained unchanged between control (CTR), SS and resilient (RES) mice. This is supported by the finding of increased ubiquitination of NRG1 in the SS mice, which indicated increased protein degradation although protein synthesis of NRG1 was constant. Moreover, overexpression of Nedd4l in mouse mPFC led to vulnerability to stress following subthreshold social defeat stress (SSDS), while downregulation of Nedd4l expression or overexpression of NRG1 (OE-NRG1) in the mPFC rescued the vulnerability to stress-induced social avoidance and anhedonia. Therefore, we speculated that Nedd4l downregulates NRG1 protein level through the ubiquitin-mediated proteolysis system in SS mice subjected to CSDS, finally leading to depression-like phenotypes.

## Materials and methods

### Animals

Male CD-1 retired breeder mice and C57BL/6 J mice, 6–8 months and 8–10 weeks, respectively, were carefully kept in a dark–light cycle of 12 h each, during the course of the experiment with free access to water and food. These two mice species were acquired from Beijing Vital River Laboratory Animal Technology Co. Ltd (Beijing, China). The animals were assigned to different experimental groups based on the litter and gender in a way that every experimental group had a similar number of siblings’ males. All the animal experimental procedures were performed under the National Institutes of Health Guide for the Care and Use of Laboratory Animals and approved by the Animal Welfare Committee of Huazhong University of Science and Technology.

### CSDS experiment

CSDS is a good procedure in terms of predictive validity for modelling the symptoms of depressive disorders^19^. For this social defeat experiment C57BL/6 J mice were used as model, and standard cages were used to house the animals with four to five mice per cage for a 1-week acclimation period. CD-1 mice were housed individually in standard mouse cages to acclimatize to the new environment for 1 week before initiating the screening process. Following this 1 week aggressive behaviour were screened for three consecutive days during inter-male social interactions (SIs) based on the criteria described previously^20^. One resident CD-1 aggressor mouse was placed overnight for habituation in one side of the divided a new home cage before the social defeat sessions start. On day 1 of SDS (social defeat sessions), the intruder mouse (C57BL/6 J) was placed directly into an equivalent side of the cage containing the aggressor mouse (CD-1) for ~5–10 min. After this period of time of social defeat, the intruder mouse was transferred to the other side of the perforated plastic divider and housed within that compartment side for the rest of the period of 24 h. Social defeat sessions were performed in novel CD-1 aggressors’ home cages daily for 10 days. For the CTR group, C57BL/6 J mouse pairs were placed in a uniform social defeat cage setup with one mouse on either of both sides of the perforated plastic divider for the 10-day duration of the social defeat test with none any physical contact. The CTR group mice were interchanged daily between CTR cages with perforated plastic divider.

### SSDS experiment

For SSDS, the mice were subjected to a subthreshold variant of the normal CSDS protocol that was used to assess increased susceptibility to stress. One C57BL/6 mouse was taken into the home cage of the aggressor CD-1 mouse for no >5 min, and allow the experimental mouse to be physically defeated by the CD-1 aggressor mouse. Following these 5 min of physical interaction, the experimental mouse went through a 20 min session of sensory stress. Subthreshold social defeat sessions during a novel CD-1 aggressors’ home cage was repeated daily for 3 days^21,22^. Once they weren’t located in aggressors’ cages, SSDS mice were singly housed in standard cages, and this protocol didn’t result in social avoidance behaviour.

### SI test

In order to assay avoidance behaviours a two-trial SI test was carried out. In the first 3 min trial, the target mouse was absent, while the test C57BL/6 mouse was left to freely explore the open-field arena (square-shaped) equipped with an empty cage placed at one side. Movements were automatically recorded with a tracking software system to work out the exploratory baseline behaviour and locomotion, while a social target is absent. A second 3 min trial was carried out this time with the target animal present, the mouse was relocated into the central filed that now contained a stranger CD-1 mouse in the same small cage, and the time spent in the interaction zone and the corner zones was tracked and compared. SI ratio was obtained by getting the ratio of the amount of time spent in the interaction zone when the target was present over that when the target was absent. All test mice with an SI ratio <1.0 were determined as SS, and everyone with an SI ratio > 1.0 was categorized as RES.

### Sucrose preference test

This test was used to evaluate anhedonia responses of stressed versus unstressed mice. Mice were allowed to freely select and drink from a bottle containing clean water, and the another containing one with 1% sucrose solution that were placed into each cage. For two consecutive days all test mice were familiarized with a 1% sucrose solution, with the positions of the bottles being interchanged every 12 h to avoid place preference. For 12 h before the test, the mice were refused access to water and then were allowed to freely drink from pre-weighed bottles for 12 h, the positions of that were interchanged during the test. The sucrose preference ratio was gotten by calculating the ratio of the ingested amount of the sucrose solution by the mouse to the total fluid intake.

### Tail suspension test

For this experiment, each mouse was suspended from a 35 cm above the floor placed horizontal bar, by the tail, and using a tape ~1 cm from the tip of the tail to acquire an immobile posture. Each test session was recorded and tracked for a period of 6 min, and the time spent immobile of every mouse was monitored and analysed during the last 4 min.

### Novelty-suppressed feeding

This test helps to evaluate the anxiety induced by the stress via assessing the degree of aversion to feeding in an open and a new environment despite prior food deprivation^23^. The mice were food-deprived overnight before testing, then a single food pellet was placed in the centre of an unfamiliar open-field arena. A mouse was then placed gently in the corner of the box and timed. The latency or time spent before eating was measured with the aid of a stopwatch. This is defined by the primary bite into the familiar food pellet for mice. Mice were observed for a maximum period of 10 min before biting into the food.

### Open field test

Mice were individually allowed to manoeuvre freely, while monitoring and tracing their locomotion in a white open-field arena (45 cm × 45 cm × 45 cm), using an automatic system (HVS Imagen, UK). The time spent by the mice in the central area (27 cm × 27 cm) during the 5 min trial period was evaluated, and the centre duration or movement distance was used to measure anxiety.

### The elevated plus maze

This test is widely used in behavioural experiments to determined anxiety behaviour^24^. Facing toward the open arm, mice were taken to the centre of the elevated plus maze and stay for 5 min in dim light conditions. A video-tracking system was employed to assess the time spent or frequency of entry in the open and closed arms.

### Stereotaxic surgery

C57BL/6 J mice were deeply anaesthetized with 6% chloralhydrate (0.6 ml/100 g i.p.) then fixed during a stereotaxic apparatus (RWD, Shenzhen, China). Bilateral microinjections of AAV9 were applied to the mouse’s PrL and IL subregions of the mPFC, at the subsequent coordinates: ±0.5 mm (M/L), + 2 mm (A/P), and −2.7 mm (D/V) relative to bregma^22^.

A total volume of 1 µl of OE-NRG1 (AAV9-Nrg1, hSyn promoter-MCS-EGFP-3FLAG-SV40 Poly A, 1.2 × 10^13^ genome copies per ml) or CTR vector was injected to every hemisphere for a period of 8 min for NRG1 overexpression experiments. The injector was left immobile within the mentioned area of the brain for at least another 8 min to prevent reuptake of injected virus as much as possible.

For the downregulation experiments of Nedd4l, a total volume of 1.2 µl of Sh-Nedd4l (AAV9-Nedd4l-RNAi, U6-MCS-CAG-EGFP, 3.69 × 10^12^ genome copies per ml) or CTR vector was infused over an 8 min period into each hemisphere. Before the CSDS, mice were allowed for 21 days to recover from the procedure and to maximize the peak expression of the viral constructs.

For Nedd4l overexpression (OE-Nedd4l) experiments, however, a total volume of 1 µl of OE-Nedd4l (AAV9-Nedd4l, hSyn promoter-EGFP-MCS-SV40 Poly A, 1.16 × 10^13^ genome copies per ml) or CTR vector was infused over an 8 min period into each hemisphere. The mice were left to recover for 28 days before SSDS to maximize the height expression of the viral constructs. All viruses were constructed by the Shanghai Genechem Co., LTD. When the behavioural testing completed, the test mice were sacrificed for a series of experiments, including neuroanatomy and gene expression.

### Western blotting and co-immunoprecipitation

Hippocampi and mPFC tissues were rapidly isolated from the brain and were homogenized at 4 °C in a lysis buffer that contains 10 mM Tris-Cl (pH 7.6), 1 mM Na_3_VO_4_, 50 mM NaF, 1 mM edetic acid, 1 mM PMSF and 1 mM benzamidine. After 10 min centrifugation of the homogenates at 4 °C and 12,000 r.p.m., the supernatants were harvested. Low RIPA lysis buffer (Beyotime) added with protease inhibitor was used to incubate the cells samples for 10 min and the lysate collected from the petri dishes on ice. Bromophenol blue and β-mercaptoethanol were added at a final concentration of 0.05% and 10%. respectively. Using a water bath samples were then boiled for 10 min for western blotting. A total of 10% SDS–polyacrylamide gel was used to carry out electrophoresis for protein separation and then transferred to a nitrocellulose membrane (Merck Millipore). Following a 1-h block in 5% non-fat milk, membranes were then incubated with primary antibodies at 4 °C overnight and accordingly with second antibodies for another 1 h at room temperature in the dark.

For co-immunoprecipitation (Co-IP), samples were incubated with protein A + G agarose at 4 °C for 5 h then centrifuged at for 3 min at 8000 r.p.m. and 4 °C. After centrifugation, the supernatants were then incubated with specific antibodies and protein A + G agarose at 4 °C overnight. Then the mixtures were again centrifuged for 10 min at 12,000 r.p.m. and 4 °C. The protein products were subsequently collected following a three times wash of the pellets with PBS, and were then mixed with loading buffer and boiled for 10 min. After centrifugation for 1 min at 12,000 r.p.m. and 4 °C, the supernatants were collected for western blotting.

The antibodies used were: NRG1, anti-NRG1 antibody (ab53104), Abcam, pAb, 1:100 for Co-IP, 1:1000 for WB; NRG1, NRG1 mAb (7D5) MA5-12896, Invitrogen, 1:100 for Co-IP, 1:500 for WB; Ubiquitin (UBI), anti-Ubiquitin antibody (ab19247), Abcam pAb, 1:1000 for WB; Nedd4l, Nedd4l antibody 4013 S, Cell Signalling, pAb, 1:1000 for WB; β-actin, anti-β actin antibody (ab6276), Abcam, mAb, 1:5000 for WB.

### Golgi staining

This experiment was conducted using an FD Rapid Golgi Stain™ Kit (FD neurotechnology, PK401). The experimental method was performed strictly consistent with the instructions of the kit^25^. Briefly, the mice were anaesthetized and perfused with 9% saline, the brains were then carefully dissected and completely submerged in an impregnation solution (containing mercuric chloride, potassium chromate and potassium dichromate) for 2 weeks in a dark environment at 4 °C. Next, the brains were moved into another solution for 72 h in the same environment. Using a vibrating microtome (Leica VT1000S; Leica, Germany), the brains were then cut into 100 μm sections, and the sections were mounted on gelatine-coated slides. After washing, the slides were dehydrated in four gradient concentrations of ethanol four times for 5 min each time, finally, visualized under an optical microscope (Olympus BX60, Tokyo, Japan).

### Immunofluorescence

For this experiment, 6% chloralhydrate was used to deeply anaesthetize the mice were followed by perfusion with 100 ml 0.9% NaCl and then by 100 ml phosphate buffer containing 4% paraformaldehyde (PFA). Brains were removed and postfixed in 4% PFA for 24 h, and then cryoprotected in 30% sucrose solutions. After the brains completely sunk in the 30% sucrose brain samples were chopped into 30 mm sections using a cryostat (CM1900, Leica). The frozen brain sections were washed in PBS carefully and permeabilized with 0.5% Triton X-100 for 30 min, then incubated in 3% BSA to block nonspecific binding for 1 h. The brain slices were incubated with NRG1 and Nedd4l primary antibodies at 4 °C overnight (1:200 dilution in 0.3% PBST for all antibodies). After three washes in PBS, brain sections were incubated with secondary antibodies for 1.5 h (1:250 dilution in 0.3% PBST for all antibodies) at room temperature. DAPI (1:1000 in PBS) was used to stain the nuclei. The images were captured employing a laser two-photon confocal microscope (LSM710, Zeiss, Germany).

### RNA extraction and quantitative real-time PCR

The TRIzol Reagent method (CWBIO, WUHAN) was used to extract the total RNA from the brain tissue and cells. About 50 mg extracted RNA was used to generate first-strand complementary DNA using the ReverTra Ace qPCR RT kit (TOYOBO, USA). A duplicate of standard polymerase chain reaction (PCR) mixture was prepared using SYBR Premix Ex Taq (TaKaRa, Dalian, Japan) to perform quantitative PCR (qPCR). These samples were evaluated on a QuantStudio 12 K Flex Real-Time PCR System. The levels of mRNA of interested genes were normalized by GAPDH mRNA. The qPCR primer sequences were as follows: sense: 5′-ATGGTGAAGGTCGGTGTG-3′ and antisense: 5′-CATTCTCGGCCTTGACTG-3′ used for GAPDH; sense: 5′- ACCAGCCATCTCATAAAGTGCG-3′ and antisense: 5′-TTGACGGGTTTGACAGGTCC-3′ used for NRG1; sense: 5′-CCTCGGAATCAGACAATAACATCAG-3′ and antisense: 5′-GCAACCTTCGGCTCACTTCT-3′ used for Nedd4l.

### Primary cortical neuron culture

For the cortical primary neuron culture, the neuronal cells were isolated from E17 to E18 Sprague–Dawley rat embryos. Cortical neurons were isolated and placed into ice-cold D-Hank’s-buffered saline in 0.25% trypsin and incubated for 15 min at 37 °C. The tissue was then gently triturated 20–30 times using a pipette for the cells to be dissociated and obtain a homogenous cell suspension. Neurons were seeded at a density of 1 × 10^5^ cells per well onto previously poly-D-lysine (25 μg/mL) coated six-well plate containing F-12 medium with 10% FBS. After 4–5 h, the planting medium was gently replaced with 1.5 ml of fresh maintenance medium containing 96% neurobasal medium, 1% GlutaMAX, 2% B-27, and 1% penicillin and streptomycin and cultivated at 37 °C cell incubator. All primary cortical neuron culture related reagents were purchased from Thermo Fisher Scientific. Every 3–4 days half of the media was replaced. The primary cortical neurons were cultured for 10 days in a humidified 5% (vol/vol) CO_2_ incubator at 37 °C, then 0.5 µM dexamethasone (Dex, Sigma) or vehicle was applied twice each day for next 10 days after 10 days in culture. The cultured primary cortical neurons were cultured with 10 μM MG132 (a selective proteasome inhibitor, Calbiochemicon) for 12 h or 10 μg/ml cycloheximide (CHX, a protein synthesis inhibitor, Absin) for 4 h, 8 h or 12 h in DIV 20 (day 20 in vitro). After treatment with Dex 1 µl/ml Si-Nedd4l virus (LV-Nedd4l-RNAi, hU6-MCS-Ubiquitin-EGFP-IRES-puromycin, 9.0 × 10^8^ genome copies per ml) was added to the cultured primary cortical neurons, then the cells were lysed and collected according to the previous method for further study.

### RNA sequencing and analysis

The mPFC tissues of CTR mice, SS mice and RES mice were rapidly isolated from the brains (three biological replicates for each group) on ice and for RNA-seq experiments were performed by Novogene (Beijing, China), then differential pathways associated with protein degradation were selected for KEGG pathway analysis (NovoMagic v3.0). All differentially expressed genes were determined by |log_2_FoldChange| > 0 and *p*-value < 0.05.

### Quantification and statistical analysis

In vitro assays, experiments were conducted by other one that don’t know the sample number and groups. In vivo assays the animal’s IDs were decoded in any case samples were analysed. Data were represented as the mean ± SEM and analysed by the statistical software GraphPad Prism 8.0 for windows. Student’s *t*-test was used to assess differences between two groups, and one-way or two-way ANOVA were used to evaluate differences among three or more groups, respectively. Statistical significance was deem accepted at *p* < 0.05.

## Results

### CSDS induces depression accompanied with an increase in NRG1 degradation

**Fig. 1:**
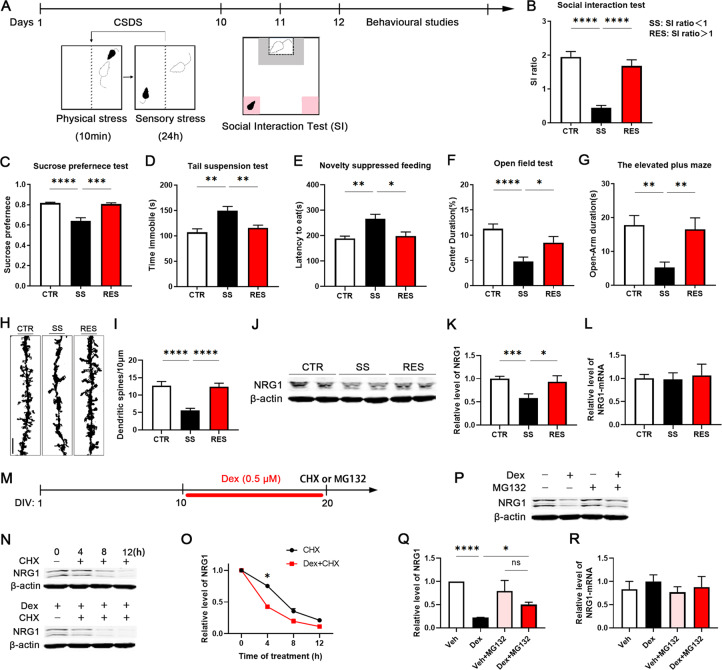
CSDS induces depression accompanied by an increase in NRG1 degradation. **a** Experimental timeline of CSDS, SI, behavioural studies and tissue collection. CSDS-induced depression-like behaviours as assessed by **b** SI test, **c** the sucrose preference test and **d** the tail suspension test, and anxiety-like behaviours as assessed by **e** novelty-suppressed feeding, **f** the open field test and **g** the elevated plus maze test, *n* = 12/12/8. **h**, **i** Golgi staining was performed to evaluate changes in dendritic spines in the mPFC between CTR, SS and RES mice, and at least ten neurons from three mice per group were analysed. Scale bars, 10 μm. **j**–**l** NRG1 protein level (*n* = 8/8/4) was lower in the mPFC of SS mice. There were no differences in NRG1 mRNA levels (*n* = 9/8/6) between CTR, SS and RES mice. **m**–**o** Experimental timeline of treatment with dexamethasone (Dex), cycloheximide (CHX) and MG132 in primary cortical neurons. Primary cortical neurons were cultured in vitro for a total of 20 days in vitro, including treatment with 0.5 µM Dex twice a day from DIV 10 to DIV 20. Western blot was used to determine levels of NRG1 in primary neurons treated with 10 μg/ml CHX for 4 h, 8 h or 12 h in DIV 20, *n* = 3 per group. **p**–**r** Inhibition of proteasomes by treatment with 10 μM MG132 for 12 h of cultured primary neurons induces NRG1 elevation with Dex treatment for 10 days as measured by western blotting. There were no differences in NRG1 mRNA levels among all groups, *n* = 3 per group. *p*-Value significance is calculated from a one-way ANOVA or two-way ANOVA. *p < 0.05, **p < 0.01, ***p < 0.001, ****p < 0.0001. All data represent mean ± SEM.

The neurobiology of chronic stress is key to the understanding of a myriad of human mood disorders^26^. Social defeat is a source of chronic stress affecting normal brain physiology and behaviour. Loss of NRG1 within the cortical projection neurons led to impaired synaptic plasticity, while chronic stress could lead to decreases in spine density and dendrite complexity in the prefrontal cortex. We therefore here investigated the effect of CSDS, an ethologically consensus model of depression in mice (Fig. 1a), on the role of NRG1 in depression-like behaviours.

A clinical diagnosis of depression is formed after the person displays variety symptoms, including social avoidance behaviour, hopelessness, self-reported depressed mood, anhedonia, sleep disturbances, psychomotor alterations, weight changes and suicidal thoughts. To test the impact of NRG1 on depression-like behaviours, we observed CSDS mice to identify individual peculiarities in the neurobiology mechanisms that might potentially underlay chronic stress responses. CSDS leads to a depression-like phenotype in a subset of mice termed SS mice. Defeated mice that do not show social avoidance as measured by the SI test are considered RES, and there was no physical contact for the CTR group^27^. After 10 days of the experiment, 12 (60%) from a total of 20 defeated mice showed SS behaviour (Fig. 1a, b, Supplementary Fig. 1a, b). The weights of the defeated mice began to decrease significantly during the past 4 days of the CSDS procedure (Supplementary Fig. 1c), and the CSDS procedure also reduced the sucrose preference of SS mice (Fig. 1c). It had been observed that SS mice had longer immobility time than the CTR and RES mice in an alternate acute stress assay, the tail suspension test (Fig. 1d). In the novelty-suppressed feeding test, a model in which increased latency to feed is associated with stress-induced anxiety, SS mice displayed significantly increased latency (Fig. 1e). Compared with CTR and RES mice, in an open field test, SS mice exhibited lower exploratory activity, as suggested by decreased time spent and distance travelled in the centre (Fig. 1f, Supplementary Fig. 1d–g). The elevated plus maze test as an assessment of anxiety-like behaviour revealed that SS mice spent less time in, and displayed a lower number of entries into, the open arms (Fig. 1g, Supplementary Fig. 1h–k). These results suggest that the CSDS procedure in mice is a validated and stress-related model of depression, which induces physiological and behavioural changes that recapitulate depression- and anxiety-like symptomatology.

It has been shown that synaptic deficiency in the prefrontal cortex is associated with depression^3^, we thus here investigated the possible mechanism based on morphology. Golgi staining showed a significantly reduced dendritic spine density in SS mice compared with CTR and RES mice (Fig. 1h, i). These findings suggest that CSDS-induced behavioural impairments are associated with synaptic dysfunction.

Loss of NRG1 has been related to impaired synaptic plasticity that is in turn associated with chronic stress and depression. We then investigated the alteration in NRG1 expression after CSDS in brain regions, like mPFC, that are associated with depression. Our findings revealed that NRG1 level was obviously decreased in the mPFC of SS mice compared with CTR and RES mice (Fig. 1j, k), although it was unchanged in hippocampus (Supplementary Fig. 1l, m). However, the NRG1 mRNA level did not differ in the mPFC or hippocampus between the CTR, SS and RES mice (Fig. 1l, Supplementary Fig. 1n). To elucidate this result, we speculated that NRG1 degradation in SS mice is increased, although the protein synthesis of NRG1 was constant. To address this issue, we used Dex treatment to mimic chronic stress^28,29^. Primary cortical neurons were cultured for a total of 20 days in vitro, including treatment with 0.5 µM of Dex twice each day from DIV 10 (day 10 in vitro) to DIV 20 (day 20 in vitro). We found that Dex treatment accelerated NRG1 degradation, as suggested by more decreased NRG1 in Dex-treated primary neurons than the vehicle CTR group after treatment for 4 h, 8 h or 12 h with CHX (Fig. 1m–o). To further confirm that the decrease in NRG1 protein level results from proteasomal degradation, we employed MG132 after the Dex downregulation of NRG1 in primary neurons^15^. We found MG132 treatments induced NRG1 accumulation, but did not affect the level of NRG1 mRNA (Fig. 1p–r). Moreover, the protein level of NRG1 was higher in the Dex and MG132-treated neurons than the Dex only treated ones (Fig. 1p, q). Taken together, the findings indicate that NRG1 degradation occurs primarily through the proteasomal pathway, resulting in a decrease in NRG1 protein levels in chronic stress cells (Dex) and the mouse (CSDS) models.

### OE-NRG1 attenuates CSDS-induced depression-like behaviours

**Fig. 2:**
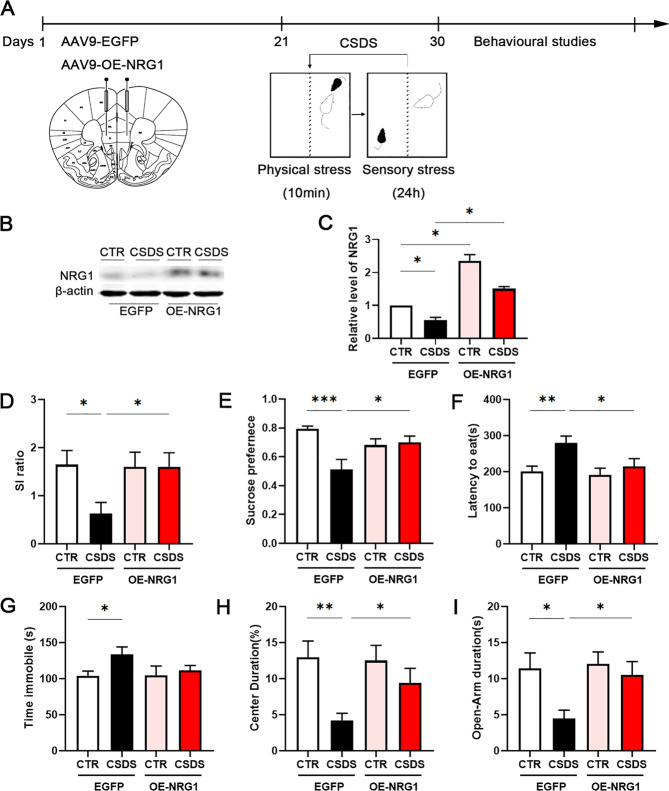
Overexpression of NRG1 attenuates CSDS-induced depression-like behaviours. **a** Experimental timeline of mPFC bilateral injection of AAV9-EGFP or AAV9-OE-NRG1 viruses and behavioural studies. **b**, **c** Representative western blots and quantification of NRG1 protein in the mPFC of non-stressed (CTR) mice, and mice subjected to CSDS after bilateral injection of AAV9-EGFP or AAV9-OE-NRG1 viruses, *n* = 3 per group. Overexpression of NRG1 before CSDS-induced depression-like behaviours as assessed by **d** social interaction, **e** sucrose preference, **f** novelty-suppressed feeding, **g** tail suspension test, **h** open field test and **i** the elevated plus maze test, *n* = 10/7/10/8. *p*-Value significance is calculated from a one-way ANOVA or two-way ANOVA. *p < 0.05, **p < 0.01, ***p < 0.001. All data represent mean ± SEM.

We have found that NRG1 level was downregulated in the mPFC of the SS mice group. To further investigate whether the loss of NRG1 in the mPFC neurons was necessary or liable for the increased susceptibility to stress, NRG1 was overexpressed by bilateral microinjection with viruses into the PrL and IL subregions of mPFC(Fig. 2a). After 3 weeks, robust expression of AAV9-hSyn-NRG1-EGFP was confirmed by western blotting in mice with and without being subjected to CSDS (Fig. 2a–c). We next evaluated whether OE-NRG1 could attenuate CSDS-induced depression-like behaviours. Several behavioural tests were performed, and therefore the result showed that NRG1 overexpression in the mPFC attenuates the CSDS-induced social avoidance and thereby promotes resilience, as indicated by significantly longer time spent in the interaction zone and lesser time spent in the corners compared to the social defeated AAV9-hSyn-EGFP CTR mice (Fig. 2d, Supplementary Fig. 2a, b). Defeated OE-NRG1 mice demonstrated increased sucrose preference and decreased latency time to feed in the sucrose preference test and the novelty-suppressed feeding test respectively, compared to defeated AAV9-EGFP mice (Fig. 2e, f). AAV9-EGFP mice and OE-NRG1 mice subjected to CSDS did not show significant differences in immobility time in the tail suspension test (Fig. 2g). The open field test, which measures activity and anxiety-like behaviour (decreased time spent in the centre), showed that defeated OE-NRG1 mice were more active than defeated AAV9-EGFP mice and spent more time in the centre of the chamber, suggesting reduced anxiety (Fig. 2h, Supplementary Fig. 2c–f). Moreover, the elevated plus maze test revealed that defeated OE-NRG1 mice spent more time in, and more frequently entered into, the open arms than the defeated AAV9-EGFP mice (Fig. 2i, Supplementary Fig. 2g–j). These results suggest that loss of NRG1 is at least partially responsible for the stress susceptibility seen in defeated mice that NRG1 overexpression in the mPFC neurons attenuates stress-induced depression- and anxiety-like behaviours.

### RNA sequencing data indicate that Nedd4l is significantly increased in SS mice

**Fig. 3:**
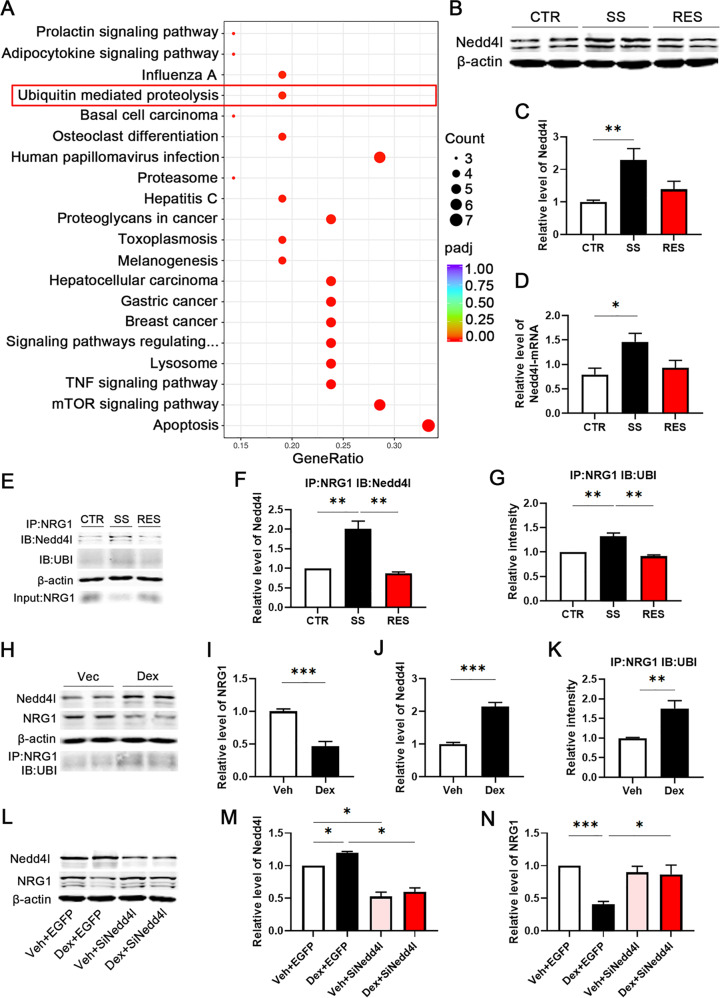
The interaction between Nedd4l and NRG1 promotes the ubiquitination and degradation of NRG1. **a** Scatter plot of KEGG pathway enrichment from RNA-seq data showed significant changes related to protein degradation in the CTR and SS mice. Highlighted by the red box is the ubiquitin-mediated proteolysis pathway, in which we were most interested. **b**–**d** Mice subjected to CSDS showed elevated Nedd4l protein levels in the medial prefrontal cortex (mPFC) of SS mice as measured by western blotting, *n* = 6 per group. The mRNA level of Nedd4l was enhanced in the mPFC samples of SS mice, and the same result was found with RNA-seq, *n* = 4/5/3. **e**–**g** The mPFC tissue homogenates were immunoprecipitated (IP), using anti-NRG1 and anti-Nedd4l antibodies, *n* = 4 per group; NRG1 ubiquitination was analysed by western blotting and quantification in CTR, SS and RES mice, *n* = 3 per group. **h**–**k** Representative western blots and quantification of NRG1, Nedd4l and NRG1 ubiquitination (UBI) identified in primary cortical neurons cultured in vitro for a total of 20 days, including treatment with 0.5 µM Dex from DIV 10 to DIV 20, *n* = 4 per group, two-tailed Student’s *t*-test. **l**–**n** Representative western blots and quantification of NRG1 and Nedd4l identified in primary cortical neurons cultured for 20 days, and treated with 1 µl/ml LV-Nedd4l-RNAi virus after treatment with Dex, *n* = 3 per group. *p*-Value significance is calculated from a one-way ANOVA. *p < 0.05, **p < 0.01, ***p < 0.001. All data represent mean ± SEM.

Considering that altered gene expression has previously been linked to resiliency or susceptibility to depression, we performed RNA-seq on the mPFC of CSDS mice and investigated the protein degradation pathway in CSDS mice. A comparison of differentially expressed genes analysed by RNA-seq between CTR, SS and RES mouse brain samples revealed 670 upregulated genes and 462 downregulated genes within the mPFC (Supplementary Fig. 3a). A heatmap cluster analysis of differentially expressed genes by RNA-seq analysis in the mPFC samples of CTR, SS and RES mice (Supplementary Fig. 3b), and a scatter plot of KEGG pathway enrichment from RNA-seq data showed significant changes associated with protein degradation in the CTR and SS mice, including the ubiquitin-mediated proteolysis, proteasomes, lysosomes, the mTOR signalling pathway, the apoptosis pathway and others. We found differences in ubiquitin-mediated proteolysis via KEGG pathway enrichment, included the E3 ubiquitin ligase Nedd4l that was found to be upregulated in SS mice (Fig. 3a, Supplementary Table 1). We further verified the protein and mRNA levels of Nedd4l in the mPFC samples of CSDS mice and found out that the protein level of Nedd4l in SS mice was increased compared to CTR mice (Fig. 3b, c), and therefore the mRNA levels of Nedd4l, as well, were according to the RNA-seq results (Fig. 3d). These results indicate that Nedd4l was significantly increased in the SS mice and could be related to NRG1 degradation.

### The interaction between Nedd4l and NRG1 promotes the ubiquitination and degradation of NRG1

We have reported that NRG1 protein level was decreased in the SS mice, which Nedd4l, a E3 ligase, was significantly expressed in these SS mice. To examine whether Nedd4l promotes the ubiquitination and degradation of NRG1, we first evaluated the interaction between these two proteins by Co-IP, and found an increased interaction between Nedd4l and NRG1 in SS mice (Fig. 3e, f), which tightly correlated with increased NRG1 ubiquitination (Fig. 3e, g). Additionally, immunofluorescence staining showed the colocalization of Nedd4l and NRG1 in the mPFC brain region of CTR and SS mice. Representative images showed that the majority of the colocalization expression was in the cell cytoplasm (Supplementary Fig. 4). To further elucidate that Nedd4l is related to NRG1 degradation in depression, we treated primary neurons with Dex to mimic chronic stress^28^. Primary cortical neurons were cultured for a total of 20 days in vitro, including treatment with 0.5 µM of Dex twice each day from DIV 10 to DIV 20. We found that Dex treatment decreased NRG1 level (Fig. 3h, i), and at the same time increased Nedd4l level (Fig. 3j) and NRG1 ubiquitination (Fig. 3k), supporting that chronic stress induces an increase in NRG1 degradation. To further confirm that Nedd4l is required for NRG1 degradation in depression, we downregulated Nedd4l via the Si-Nedd4l virus in Dex-treated primary cortical neurons. Western blotting results (Fig. 3l) showed that the downregulation of Nedd4l prevented the decrease in NRG1 level induced by Dex treatment (Fig. 3m, n). These data together suggest that Nedd4l is significantly increased in chronic stress, and promotes the ubiquitination and degradation of NRG1.

### Overexpression of Nedd4l in the mPFC promotes stress susceptibility when subjected to SSDS

**Fig. 4:**
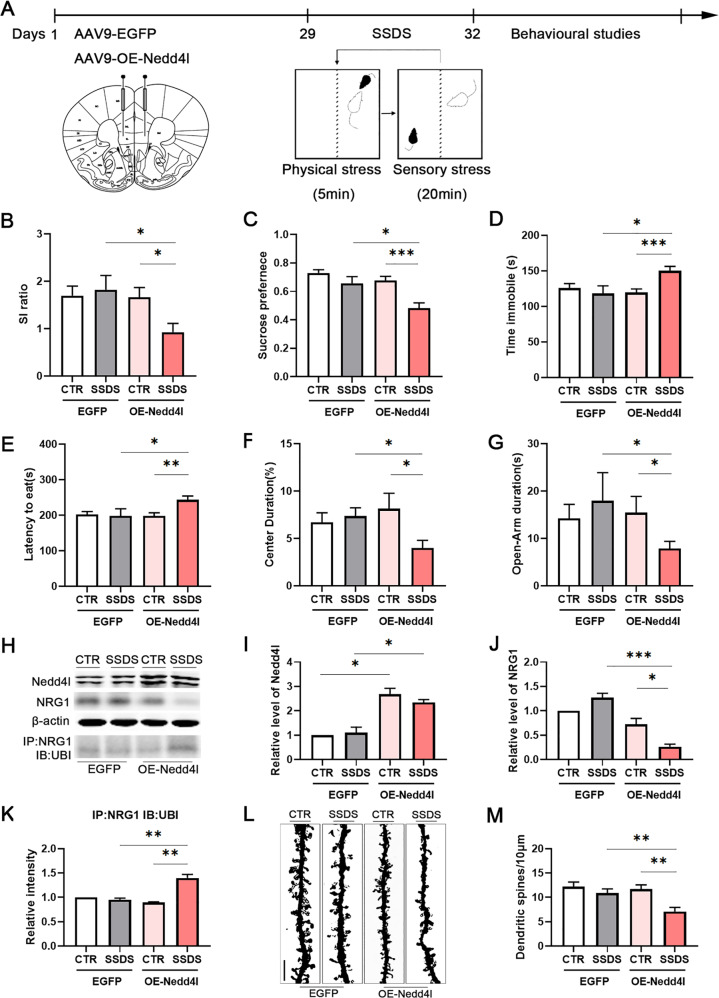
Overexpression of Nedd4l deteriorates subthreshold social defeat stress-induced depression-like behaviours. **a** Experimental timeline of mPFC bilateral injection of AAV9-EGFP or AAV9-OE-Nedd4l viruses and behavioural studies. Overexpression of Nedd4l before subthreshold social defeat stress (SSDS)-induced depression-like behaviours as assessed by **b** social interaction, **c** sucrose preference test and **d** tail suspension test, and anxiety-like behaviours as assessed by **e** novelty-suppressed feeding test, **f** open field test and **g** the elevated plus maze test, *n* = 20/9/18/22. **h**–**k** Representative western blots and quantification of NRG1, Nedd4l and NRG1 ubiquitination (UBI) identified in the mPFC of non-stressed (CTR) mice, and mice subjected to SSDS after bilateral injection of AAV9-EGFP or AAV9-OE-Nedd4l viruses, *n* = 3–4 per group. **l**, **m** Golgi staining was performed to evaluate the alterations of dendritic spines in the mPFC between CTR and SSDS mice after injection of AAV9-EGFP or AAV9-OE-Nedd4l viruses. At least ten neurons from three mice per group were analysed. Scale bars, 10 μm. *p*-Value significance is calculated from a one-way ANOVA. *p < 0.05, **p < 0.01, ***p < 0.001. All data represent mean ± SEM.

It was found during this study that stress susceptibility is related to a decreased level of NRG1. However, it is not clear whether Nedd4l regulates the expression of NRG1 in the mPFC neurons by ubiquitin-mediated proteolysis, and promotes stress susceptibility. To address this issue, Nedd4l was overexpressed through bilateral microinjection with AAV9-Nedd4l viruses into the mPFC, while AAV9-EGFP served as CTR. After 4 weeks, mice were underwent SSDS (Fig. 4a), which is a subthreshold variant of the CSDS protocol that was used to assess increased susceptibility to stress, and this procedure does not result in social avoidance behaviour. OE-Nedd4l did not alter behavioural phenotypes in mice without stress. However, subthreshold defeated mice injected with AAV9-Nedd4l showed significantly reduced SI ratios when compared to defeated AAV9-EGFP mice and OE-Nedd4l mice not subjected to SSDS, as they spent more time in the corners and less in the interaction zone when the social target was present (Fig. 4b, Supplementary Fig. 5a, b). An obvious decrease in the sucrose preference was also observed in OE-Nedd4l mice after the SSDS procedure compared to EGFP mice (Fig. 4c). Moreover, the tail suspension test (Fig. 4d) and the novelty-suppressed feeding test (Fig. 4e) showed that the subthreshold defeated OE-Nedd4l mice have longer immobility time and increased latency to feed, respectively, than the AAV9-EGFP mice and OE-Nedd4l mice not subjected to SSDS. Furthermore, the subthreshold defeated OE-Nedd4l mice showed reduced centre duration and centre distance in the open field test (Fig. 4f, Supplementary Fig. 5c–f), and spent less time in the open arm of the elevated plus maze (Fig. 4g, Supplementary Fig. 5g–j) than the AAV9-EGFP mice and the OE-Nedd4l mice not subjected to SSDS. These data strongly suggested that overexpression of Nedd4l in the mPFC promotes SSDS-induced depression- and anxiety-like behaviours in mice.

To investigate the mechanism underlying overexpression of Nedd4l mediating stress susceptibility in mice subjected to SSDS, we firstly detected the NRG1 level and its ubiquitination. We found that the expression levels of NRG1 were only reduced in the mPFC of OE-Nedd4l mice after SSDS (Fig. 4h–j), while on the other hand NRG1 ubiquitination was found to be increased only following SSDS (Fig. 4k). Next, we evaluated morphological changes by Golgi staining, which result showed that SSDS only induced dendritic spine impairments in the mPFC of OE-Nedd4l mice (Fig. 4l, m). These results suggest that OE-Nedd4l-induced ubiquitination of NRG1 decreased the expression levels of NRG1, and resulted in the deteriorated physiological and behavioural changes that recapitulate SSDS-induced depression-like behaviours.

### Knock down of Nedd4l in mPFC rescues CSDS-induced depression

**Fig. 5:**
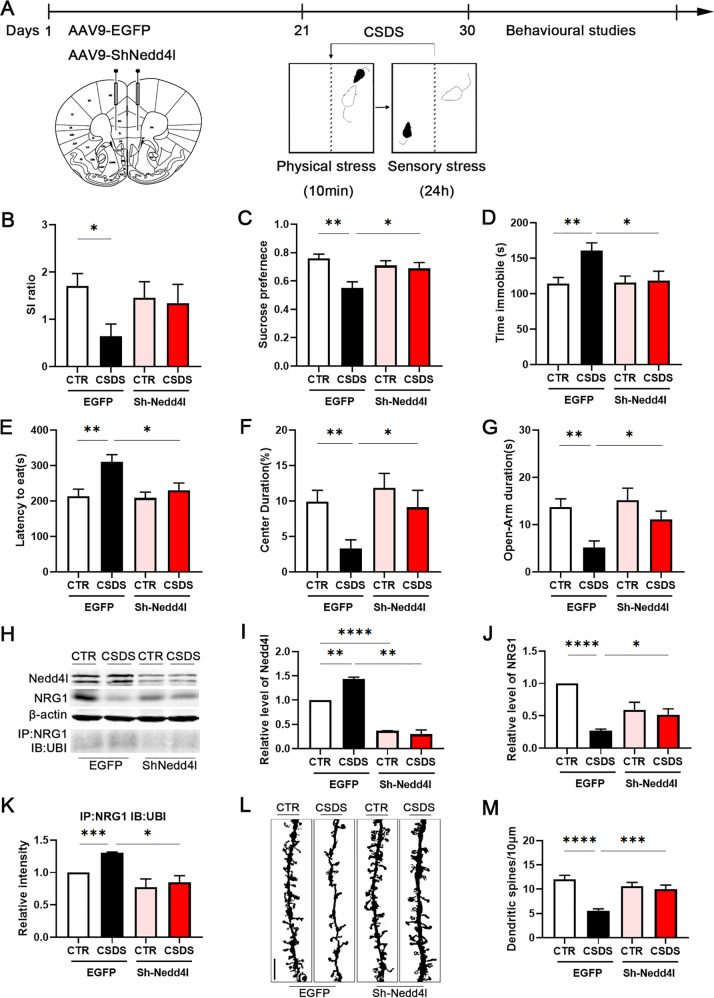
Knock down of Nedd4l rescues CSDS-induced depression. **a** Experimental timeline of mPFC bilateral injection of AAV9-EGFP or AAV9-Sh-Nedd4l viruses and behavioural studies. Downregulation of Nedd4l expression before CSDS-induced depression-like behaviours as assessed by **b** social interaction, **c** sucrose preference and **d** tail suspension tests, and anxiety-like behaviours as assessed by **e** novelty-suppressed feeding, **f** open field and **g** the elevated plus maze tests, *n* = 10/8/8/8. **h**–**k** Representative western blots and quantification of NRG1, Nedd4l and NRG1 ubiquitination (UBI) identified in the mPFC of non-stressed (CTR) mice, and mice subjected to CSDS after bilateral injection of AAV9-EGFP or AAV9-Sh-Nedd4l viruses, *n* = 3 per group. **l**, **m** Golgi staining was performed to evaluate the changes of dendritic spines in the mPFC between CTR and CSDS mice after injection of AAV9-EGFP or AAV9-Sh-Nedd4l viruses. At least ten neurons from three mice per group were analysed. Scale bars, 10 μm. *p*-Value significance is calculated from a one-way ANOVA. *p < 0.05, **p < 0.01, ***p < 0.001, ****p < 0.0001. All data represent mean ± SEM.

To determine whether Nedd4l in the mPFC neurons is required for susceptibility to stress, we firstly evaluated whether Nedd4l knock down could rescue CSDS-induced depression-like behaviours. Bilateral injections of AAV9-Sh-Nedd4l in the mPFC were used to knock down Nedd4l, while AAV9-EGFP served as CTR (Fig. 5a). After animals have recovered for 3 weeks for efficient viral expression, it had been found that Nedd4l knock-down didn’t alter behavioural phenotypes in mice without stress. However, Nedd4l knock-down in the mPFC prevented the social avoidance that was induced by CSDS, and thus promoted resilience, as indicated by the increased time spent in the interaction zone compared with the defeated AAV9-EGFP mice (Supplementary Fig. 6a). Despite a trend towards higher SI ratio and time spent in the corners, no significant differences were observed between the defeated Sh-Nedd4l mice and the defeated AAV9-EGFP mice (Figs. 5b, Supplementary Fig. 6b). Defeated Sh-Nedd4l mice demonstrated increased sucrose preference, and spent more time immobile in the sucrose preference test and the tail suspension test, respectively, compared to the defeated AAV9-EGFP mice (Fig. 5c, d). The novelty-suppressed feeding test showed that the latency to feed was markedly increased in defeated Sh-Nedd4l mice (Fig. 5e), indicating that knock down of Nedd4l in the mPFC attenuated stress-induced anxiety. Compared to defeated AAV9-EGFP mice, in the open field test, Sh-Nedd4l mice subjected to CSDS exhibited higher exploratory activity, which was indicated by the increased time spent in the centre (Figs. 5F, Supplementary Fig. 6c–f). The elevated plus maze test as a measure of anxiety-like behaviour revealed that defeated Sh-Nedd4l mice spent more time, and entered more frequently, in the open arms (Figs. 5g, Supplementary Fig. 6g–j). These findings are an indication that Nedd4l knock-down attenuated depression- and anxiety-like behaviours in mice subjected CSDS.

Next, we detected the effect of Nedd4l knock-down on the NRG1 level and its ubiquitination. The results revealed that knock-down of Nedd4l in the mPFC blocked the decrease in NRG1 level and prevented the increase in NRG1 ubiquitination in CSDS mice (Fig. 5h–k). Meanwhile, we observed that CSDS did not induce morphological impairments of dendritic spines in the mPFC of defeated Sh-Nedd4l mice, as demonstrated by Golgi staining from the brain slices of those mice (Fig. 5l, m). These data strongly support that Nedd4l knock-down increased the ubiquitination of NRG1, and rescued the physiological and behavioural changes in CSDS-induced depression-like behaviours.

Together these findings highlight a completely unique etiopathogenic mechanism of CSDS-induced depression, which is initiated by Nedd4l upregulation, promoting NRG1 ubiquitination and degradation, and facilitating synaptic impairments and CSDS-induced depression-like behaviours, therefore providing a theoretical basis for the occurrence of depression and possible intervention sites.

## Discussion

Depression results from major life stressors. It’s becoming a more serious public health problem affecting young people^2^ and is related to synaptic plasticity. The loss of NRG1 within the cortical projection neurons was reported to result in increased inhibitory connections and reduced synaptic plasticity^10,11^. Since chronic stress seems to be a visible risk factor for MDD, identifying the molecular and cellular factors underlying behavioural sensitivity or resilience to stress-induced behavioural responses is crucial to elucidating the mechanisms of MDD^30^. Here, we provide evidence that Nedd4l in the mPFC acts as a key role in the feedback of the stress response, and increases the susceptibility to stress and the effects of social defeat through the promotion of defeat-induced decreased levels of NRG1 protein in the mPFC. The findings presented in this study are the first to examine in detail the function of Nedd4l regulation of NRG1 in the mPFC brain area, and the first to identify its role in CSDS-induced depression.

Alterations in the morphology and function of the mPFC neurons are well recognized as contributing to the development of MDD^31^. We verified again that the CSDS procedure uses the social conflict among members within the same species to trigger both emotional and psychological stress. An advantage of social defeat is that it could be used to study differences among individuals in response to stress^32^. The dynamics of neuronal and behavioural changes after chronic social defeat have suggested the involvement of epigenetic factors^33^. NRG1 is a neurotrophic factor involved in numerous processes that acts by stimulating the ErbB4 tyrosine kinase^34^. NRG1 and NRG1/ErbB4 signalling regulate several processes of neurodevelopment that play extremely critical roles in neuropathology^35^. Additionally, we found that the protein level of NRG1 was reduced in the mPFC of the SS mice, but NRG1 mRNA levels were not different in either the mPFC or hippocampus between the CTR, SS and RES mice. We hence speculated that the protein synthesis of NRG1 was constant, but protein degradation might have been affected. It has been evidenced that patients with high glucocorticoid levels are more likely to experience depression recurrence^36^. Glucocorticoids bind to corticoid receptors in brain regions like the hippocampus, the prefrontal cortex and other, and play a major role in assisting individuals to adjust to and face stress and form negative feedback mechanisms^37^. In the current study, we investigated the effects of chronic stress in primary cortical neurons through treatment with the glucocorticoid receptor agonist Dex, and this suggested that NRG1 degradation primarily occurred through the proteasomal pathway, leading to a decrease in NRG1 protein levels. This was further confirmed by the treatment of the Dex-treated neurons with the protein biosynthesis inhibitor CHX or the proteasomal inhibitor MG132. Moreover, in defeated mice, NRG1 overexpression reduced sucrose preference, SI, as well as the time spent immobile in the tail suspension test. Therefore, as a measure of depression-like behaviour, decreased levels of NRG1 in the mPFC could be an important factor affecting an individual’s susceptibility to stress-induced depression and contributes to the modulation of emotional responses.

An important finding of this study was the result of RNA-seq of the mPFC of CSDS mice following social defeat, which showed significant changes related to chronic or acute inflammatory responses, natural killer cell-mediated cytotoxicity, signalling pathways regulating the pluripotency of stem cells and the apoptosis pathway, etc. To research the changes related to protein degradation in the CTR and SS mice, we analysed KEGG pathway enrichment, including ubiquitin-mediated proteolysis, proteasomes, lysosomes, the mTOR signalling pathway and the apoptosis pathway. We found that the E3 ubiquitin protein ligase Nedd4l was upregulated in SS mice via ubiquitin-mediated proteolysis, which might explain the degradation of NRG1. As a member of the Nedd4 family of ubiquitin ligases. Nedd4l is the closest homologue of Nedd4-1 (ref. ^38^). Both Nedd4-1 and Nedd4l are shown to interact with and regulate ubiquitination in certain subtypes of ion channels^39^. However, the results of the RNA-seq measurement show that only Nedd4l is upregulated while Nedd4-1 is unchanged, which may explain the difference between Nedd4-1 and Nedd4l in the CSDS of the mPFC brain area, as indicated by a significant increase in Nedd4l level in chronic stress and Nedd4l promotion of the ubiquitination and degradation of NRG1.

We next investigated whether Nedd4l in the mPFC plays an important role in promoting the behavioural and neuroendocrine phenotypes associated with stress. We found that OE-Nedd4l or knock-down did not alter behavioural phenotypes in mice without stress. However, overexpression of Nedd4l in the mPFC of subthreshold social defeat mice promoted a more vulnerable phenotype relative to the CTR AAV9-EGFP mice, with an accompanying decrease in the expression levels of NRG1. Conversely, knock down of Nedd4l in the mPFC decreased the degradation of NRG1 and rescued the behavioural changes in CSDS-induced depression-like behaviours, which strongly support that the expression of Nedd4l in the mPFC neurons is required for NRG1 deficiency-mediated susceptibility to stress. Actually, Nedd4 family members such as Nedd4l play a significant role in the substrate specificity and functions, determined by phosphorylation and the binding of adaptors and accessory proteins to specific regions of the ligase, intramolecular interactions and deubiquitination^40^. Subsequent researches are needed to explain what factors cause an increase in Nedd4l after stress and further increase the ubiquitination of NRG1.

Synaptic deficiency in the prefrontal cortex is associated with depression^41,42^. In the present study, the results also revealed a significant reduction in the dendritic spine density in the SS mice or the subthreshold defeated OE-Nedd4l mice, which are abrogated by knock down of Nedd4l in the mPFC of CSDS mice, indicating that CSDS-induced behavioural impairments are associated with, and could be the reflection of, synaptic dysfunction.

Overall, we currently explained a possible mechanism by which Nedd4l mediates depression-like behaviours by inducing NRG1 degradation following stress. The findings presented here show that the decrease in NRG1 correlates with an increase in Nedd4l in the mPFC, and promotes synaptic impairments and stress susceptibility in individuals subjected to CSDS, which, when correlated with the severity of MDD symptom, may provide the theoretical grounds for the development of treatment strategies for stress-related neuropsychologic disorders, including PTSD, anxiety and depressive disorders.

## Supplementary information

Supplementary Table

Supplementary Figure legends

Figure S1

Figure S2

Figure S3

Figure S4

Figure S5

Figure S6
