# Supplementary material for: Nedd4l downregulation of NRG1 in the mPFC induces depression-like behaviour in CSDS mice
Source: Transl Psychiatry. 2020 Jul 23;10:249. doi: 10.1038/s41398-020-00935-x (PMC7378253; doi:10.1038/s41398-020-00935-x)
Supplement: Supplementary file 1 — Supplementary Table [file 41398_2020_935_MOESM1_ESM.docx]

**Supplementary Table**

**Table S1. Different E3 ligase Expression in SS and CTR mice.**

| Gene name | SS | CTR | log2FoldChange | P value |
| --- | --- | --- | --- | --- |
| Nedd4l | 3416.1±254.0 | 2695.6±254.0 | 0.34 | 0.02 |
| Nedd4-1 | 9175.1±1246.0 | 8056.9±1246.0 | 0.19 | 0.32 |
| Ube3a | 1723.7±201.8 | 1543.7±201.8 | 0.16 | 0.37 |
| Smurf2 | 829.1±92.1 | 843.1±92.1 | -0.02 | 0.89 |
| Itch | 1151.0±118.1 | 986.0±118.1 | 0.22 | 0.19 |
| Wwp1 | 875.3±69.6 | 782.8±69.6 | 0.16 | 0.31 |
| Trip12 | 3455.8±284.0 | 3225.3±284.0 | 0.10 | 0.50 |
| Huwe1 | 7393.9±540.1 | 7244.5±540.1 | 0.03 | 0.83 |
| Ubr5 | 3504.4±190.0 | 3150.7±190.0 | 0.15 | 0.25 |
| Herc1 | 5264.5±329.1 | 4813.3±329.1 | 0.13 | 0.35 |

The mPFC tissues of SS mice and CTR mice were rapidly isolated from the brains and prepared for RNA-seq (three biological replicates for each group). All differentially expressed genes were determined by |log2FoldChange|> 0 and p-value < 0.05. SS, stress-susceptible, CTR, control.

Nedd4l, neural precursor cell expressed, developmentally down-regulated gene 4-like; Nedd4-1, neural precursor cell expressed, developmentally down-regulated 4; Ube3a, ubiquitin protein ligase E3A; Smurf2, SMAD specific E3 ubiquitin protein ligase 2; Itch, itchy, E3 ubiquitin protein ligase; Wwp1, WW domain containing E3 ubiquitin protein ligase 1; Trip12, thyroid hormone receptor interactor 12; Huwe1, HECT, UBA and WWE domain containing 1; Ubr5, ubiquitin protein ligase E3 component n-recognin 5; Herc1, HECT and RLD domain containing E3 ubiquitin protein ligase family member 1. All data represent average of the read count normalized by each sample ± SEM.
