# Supplementary material for: Nedd4l downregulation of NRG1 in the mPFC induces depression-like behaviour in CSDS mice
Source: Transl Psychiatry. 2020 Jul 23;10:249. doi: 10.1038/s41398-020-00935-x (PMC7378253; doi:10.1038/s41398-020-00935-x)
Supplement: Supplementary file 2 — Supplementary Figure legends [file 41398_2020_935_MOESM2_ESM.docx]

**Supplementary Figure legends**

**Figure S1 Social stress vulnerability is associated with reduced NRG1 expression** **in the mPFC, but not in the hippocampus.**

**(A, B)** SS mice spent less time in the interaction zone and more time in the corners when the social target was present when compared to CTR and RES mice. n = 12/12/8. **(C)**The weight of defeated mice began to decrease significantly during the last 4 days of the CSDS. n = 12/20. **(D-G)** Representative maps showing time spent in the centre and distance in the open field test, including reduced centre duration and centre distance (%). There were no differences in speed between each group. **(H-K)**Representative maps showing time spent in the open and closed arms of the EPM, including decreased time spent in the open arms and a reduced number of entries into the open arms. There were no differences in the closed arms duration between each group. n = 12/12/8. **(L-N)** There were no differences in NRG1 protein and mRNA levels in the hippocampus of CTR, SS and RES mice. n = 4 per group. P value significance is calculated from a one-way ANOVA or two-way ANOVA. *p＜ 0.05, **p＜ 0.01, ***p＜ 0.001, ****p＜ 0.0001. All data represent mean ± SEM.

**Figure S2 Overexpression of NRG1 in the mPFC rescue depression-like behaviours.**

**(A, B)** Mice injected with AAV9-OE-NRG1 in the mPFC subjected to CSDS spent more time in the interaction zone and less time in the corners when the social target was present compared to the AAV9-EGFP mice subjected to CSDS. **(C-F)** Representative maps of AAV9-OE-NRG1 mice subjected to CSDS, showing time spent in the centre and distance in the open field test compared to the AAV9-EGFP mice subjected to CSDS, including increased centre duration and centre distance (%). There were no differences in speed between groups. **(G-J)** Representative maps of AAV9-OE-NRG1 mice subjected to CSDS, showing time spent in the open and closed arms of the EPM compared to the AAV9-EGFP mice subjected to CSDS, including increased time spent in the open arms and an increased number of entries into the open arms. There were no differences in duration in the closed arms between each group. n = 10/7/10/8. P value significance is calculated from a one-way ANOVA. *p＜ 0.05, **p＜ 0.01, ***p＜ 0.001. All data represent mean ± SEM.

**Figure S3 Differentially expressed genes by RNA-seq analysis.**

**(A)**Volcano map of differentially expressed genes by RNA-seq analysis from mPFC between CTR and SS mice. Differentially expressed genes were identified with the criteria of more than 2-fold change and p-value < 0.05. **(B)** Heatmap cluster analysis of differentially expressed genes by RNA-seq analysis in mPFC samples of CTR, SS and RES mice. n = 3 mice for each group.

**Figure S4 The colocalization of NRG1 and Nedd4l.**

Representative images showing the colocalization of NRG1 (red), Nedd4l (green) and DAPI (blue) in mPFC brain region of CTR and SS mice. Scale bars, 10 μm.

**Figure S5 Overexpression of Nedd4l in mPFC induces depression-like behaviours in SSDS.**

**(A)** Mice injected with AAV9-OE-Nedd4l in the mPFC subjected to subthreshold social defeated stress (SSDS) spent less time in the interaction zone and more time in the corners **(B)** when the social target was present compared to the AAV9-EGFP mice subjected to SSDS. **(C-F)** Representative maps of AAV9-OE-Nedd4l mice subjected to SSDS showing time spent in the centre and distance in the open field test compared to the AAV9-EGFP mice subjected to SSDS, including reduced centre duration and centre distance (%). There were no differences in speed between groups. **(G-J)** Representative maps of AAV9-OE-Nedd4l mice subjected to SSDS showing time spent in the open and closed arms of the EPM compared to the AAV9-EGFP mice subjected to CSDS, including decreased time spent in the open arms and a reduced number of entries into the open arms. There were no differences in duration in the closed arms between groups. n = 20/9/18/22. P value significance is calculated from a one-way ANOVA. *p＜ 0.05, **p＜ 0.01, ***p＜ 0.001. All data represent mean ± SEM.

**Figure S6 Knock down of Nedd4l in the mPFC rescue depression-like****behaviours.**

**(A, B)** Mice injected with AAV9-Sh-Nedd4l in the mPFC subjected to CSDS spent more time in the interaction zone and no differences in the corners when the social target was present compared to the AAV9-EGFP mice subjected to CSDS. **(C-F)** Representative maps of AAV9-Sh-Nedd4l mice subjected to CSDS showing time spent in the centre and distance in the open field test compared to the AAV9-EGFP mice subjected to CSDS, including increased centre duration but not centre distance (%). There were no differences in speed between groups. **(G-J)** Representative maps of AAV9-Sh-Nedd4l mice subjected to CSDS showing time spent in the open and closed arms of the EPM compared to the AAV9-EGFP mice subjected to CSDS, including increased time spent in the open arms and increased number of entries into the open arms. There were no differences in duration in the closed arms between groups. n = 10/8/8/8. P value significance is calculated from a one-way ANOVA. *p＜ 0.05, **p＜ 0.01, ***p＜ 0.001. All data represent mean ± SEM.
