# Supplementary figures and images for: Nedd4l downregulation of NRG1 in the mPFC induces depression-like behaviour in CSDS mice
Source: Transl Psychiatry. 2020 Jul 23;10:249. doi: 10.1038/s41398-020-00935-x (PMC7378253; doi:10.1038/s41398-020-00935-x)

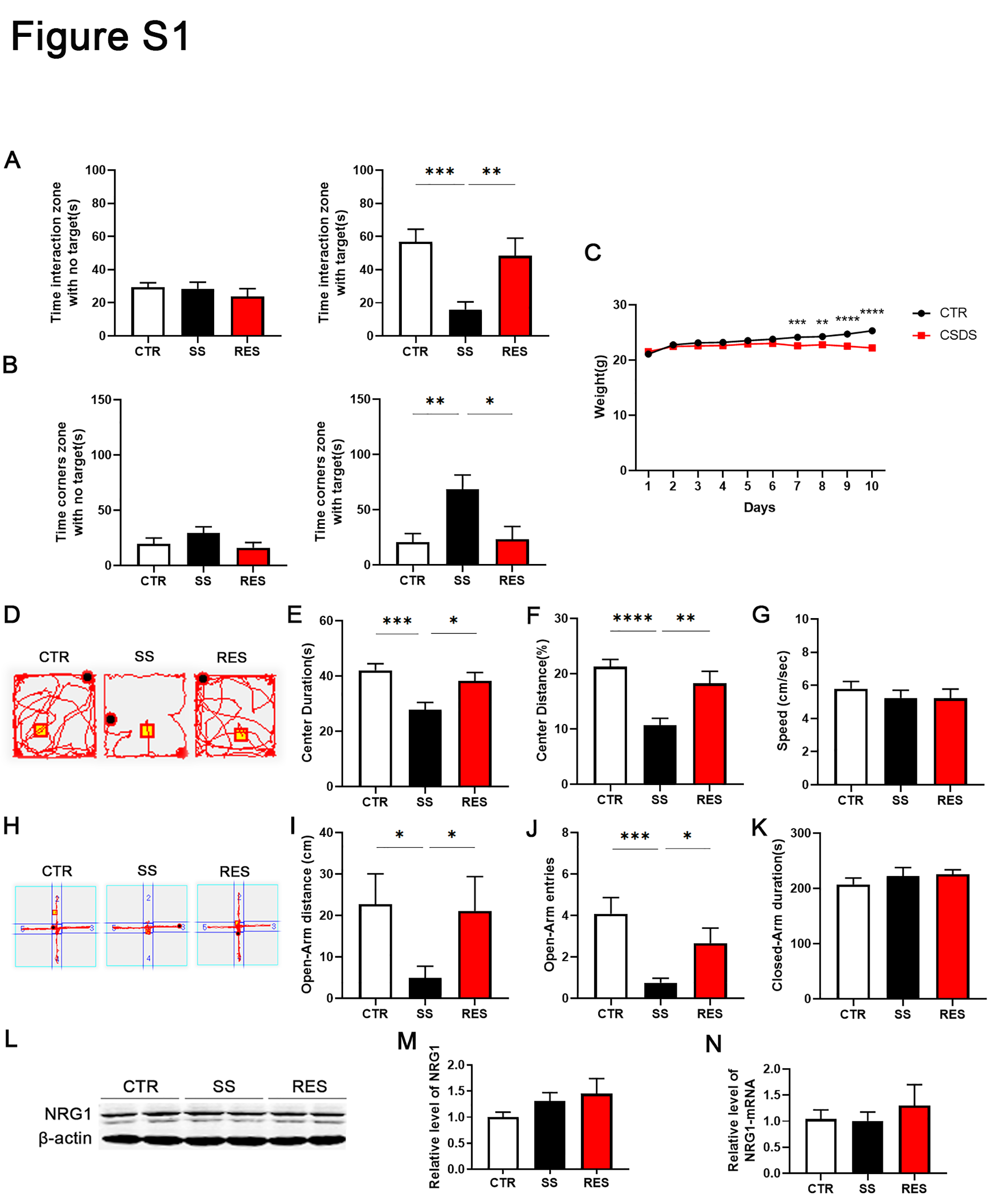

Supplement: Supplementary file 3 — Figure S1 [file 41398_2020_935_MOESM3_ESM.tif]

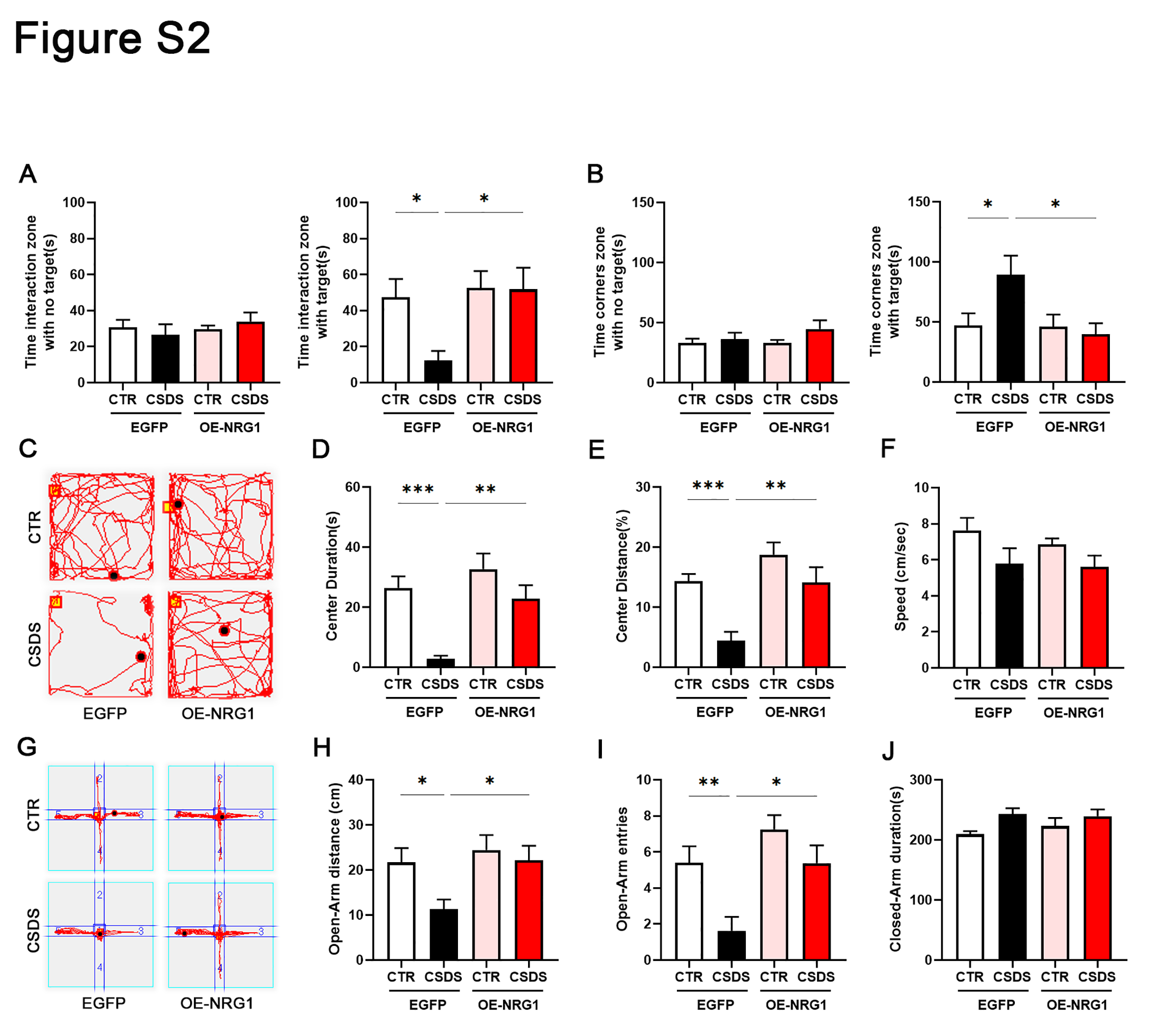

Supplement: Supplementary file 4 — Figure S2 [file 41398_2020_935_MOESM4_ESM.tif]

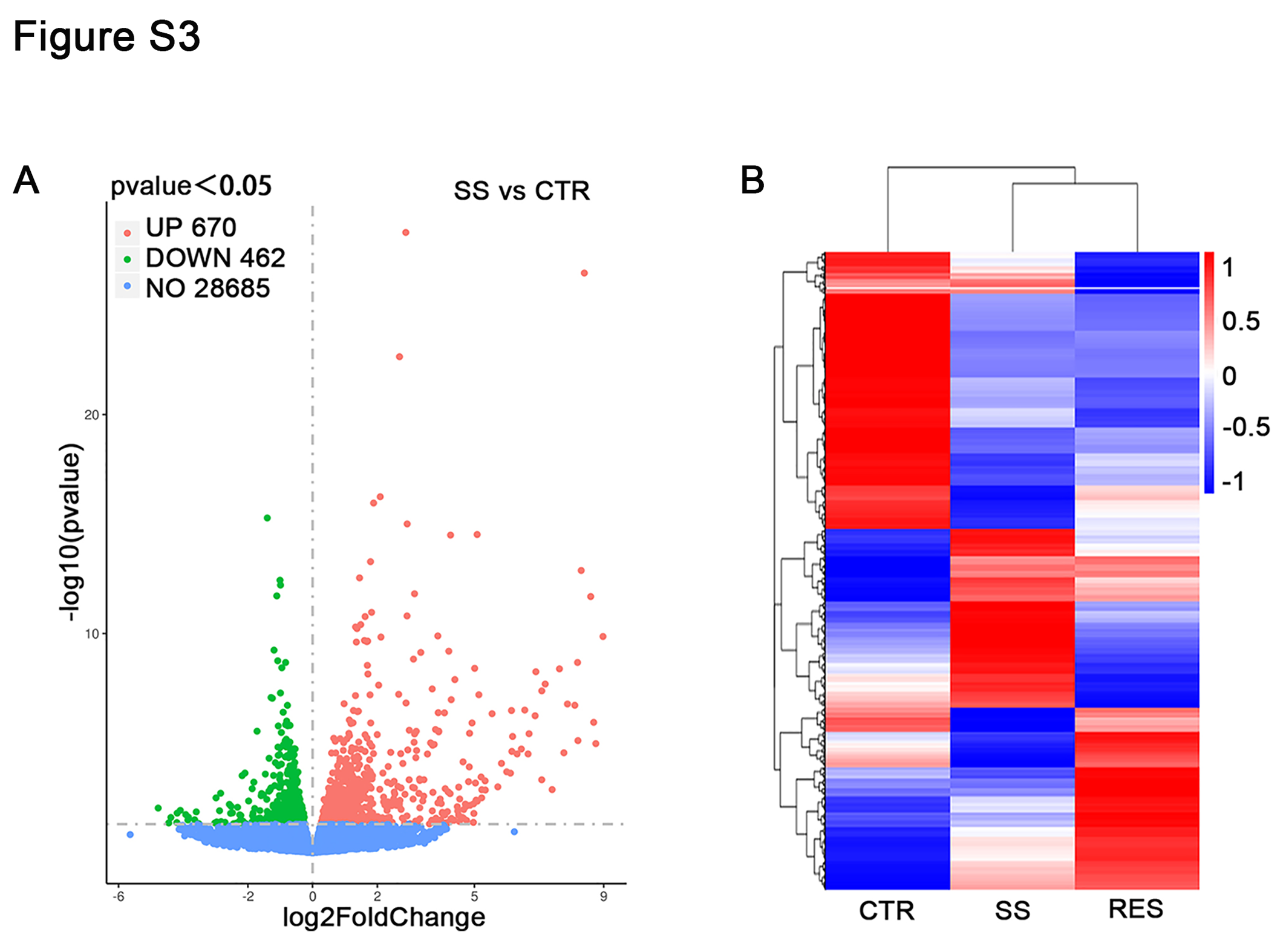

Supplement: Supplementary file 5 — Figure S3 [file 41398_2020_935_MOESM5_ESM.tif]

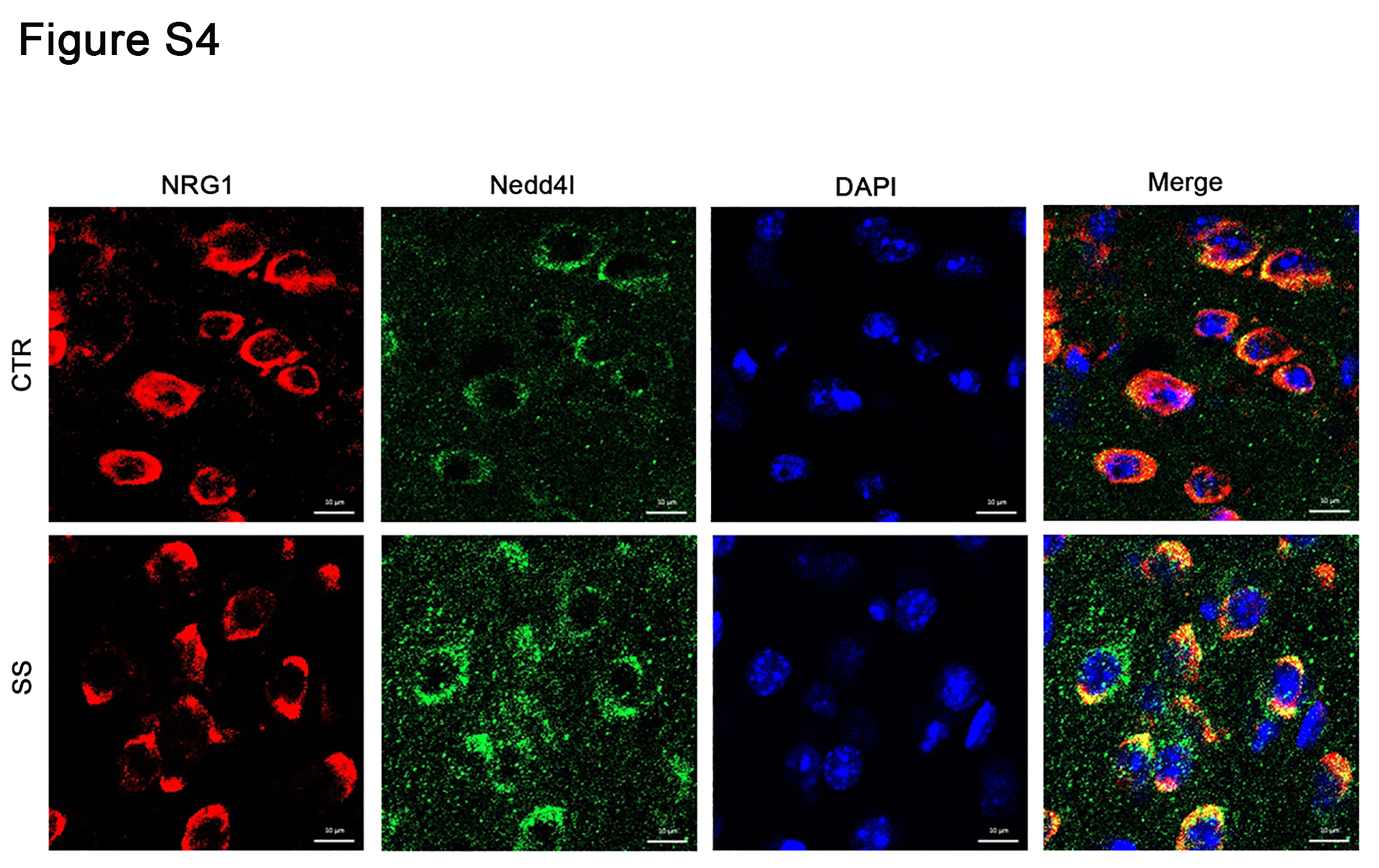

Supplement: Supplementary file 6 — Figure S4 [file 41398_2020_935_MOESM6_ESM.tif]

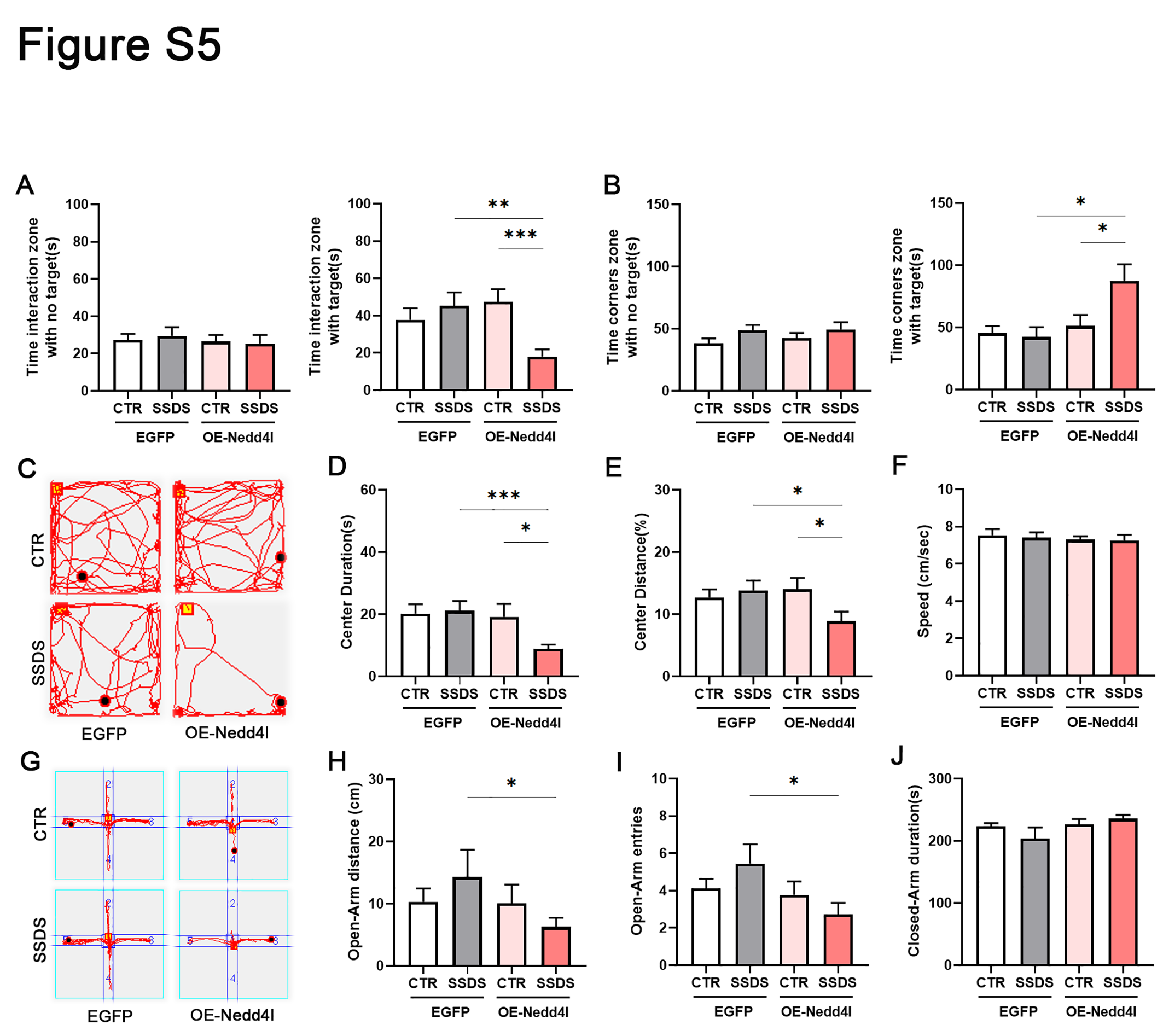

Supplement: Supplementary file 7 — Figure S5 [file 41398_2020_935_MOESM7_ESM.tif]

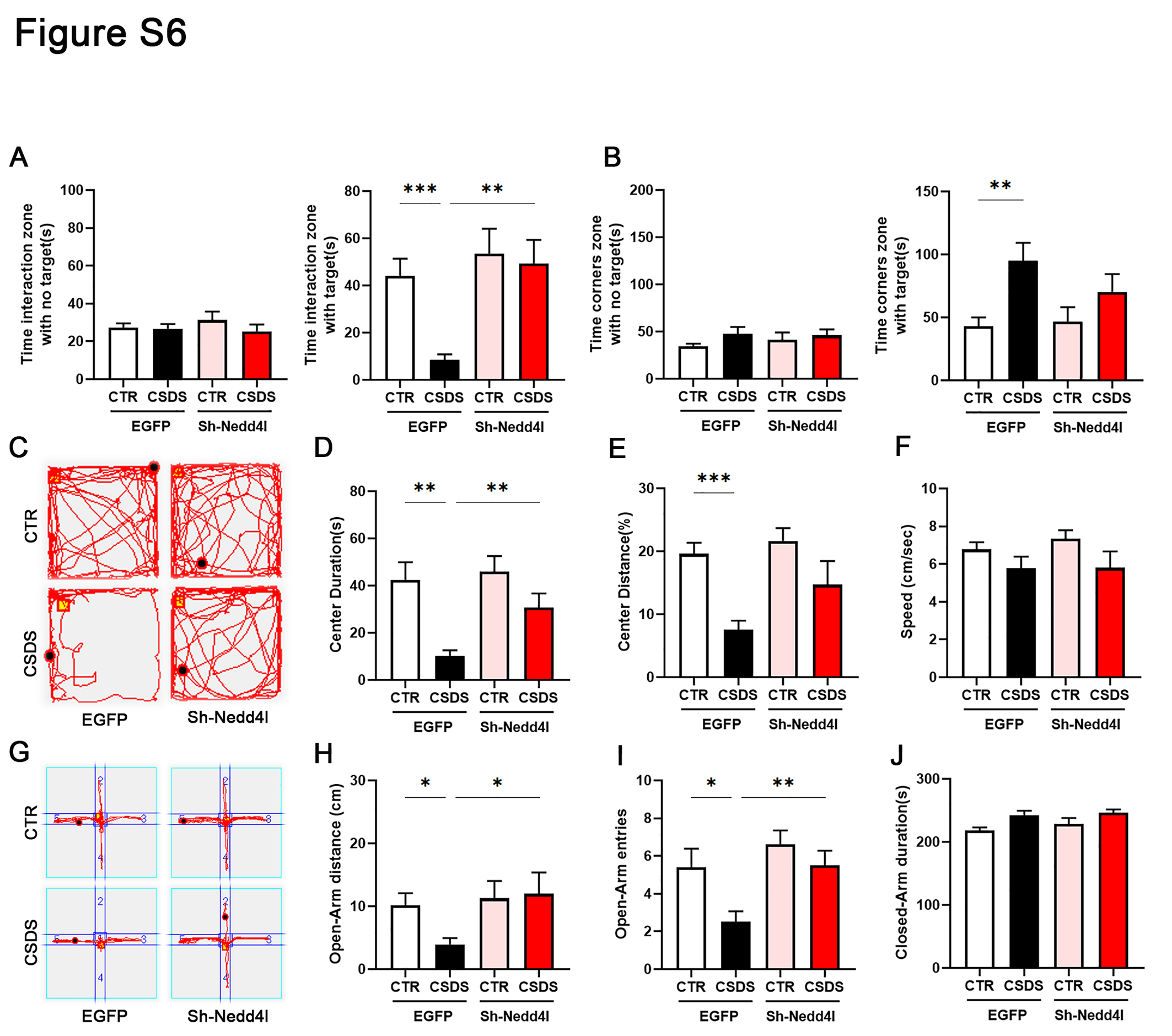

Supplement: Supplementary file 8 — Figure S6 [file 41398_2020_935_MOESM8_ESM.tif]
